# Karyotype and DNA-Methylation Responses in Myelodysplastic Syndromes following Treatment with Traditional Chinese Formula Containing Arsenic

**DOI:** 10.1155/2012/969476

**Published:** 2012-10-16

**Authors:** Sun Shuzhen, Ma Rou, Hu Xiaomei, Yang Xiao-hong, Xu Yong-gang, Wang Hongzhi, Yang Xiu-Peng

**Affiliations:** National Hematological Medical Center of Traditional Chinese Medicine, Department of Hematology, Xiyuan Hospital, China Academy of Chinese Medical Sciences, Beijing 100091, China

## Abstract

We have previously shown that arsenic-containing Chinese herbal formula, Qing-Huang powder capsule (containing tetraarsenic tetrasulfide, As_4_S_4_), is effective in the treatment of myelodysplastic syndrome (MDS); yet the underlined mechanisms remain unclear. In this study, using standard cytogenetic analysis (G-banded) and global DNA methylation method (ChIP-on-chip assays), we aimed to determine the effect of arsenic-containing Chinese herbal formula on karyotype status and the genomic methylation level in primarily diagnosed MDS patients. Correlation of aberrant DNA methylation and chromosome aberrations in MDS was also investigated. We found that the number of genes with aberrant DNA methylation was highest in MDS patients with normal karyotype, followed by trisomy 8 karyotype, and relatively low in patients with cytogenetic abnormalities other than trisomy 8. Treatment with arsenic-containing Chinese herbal formula had no effects on karyotype status, but resulted in a significant genome-wide demethylation. Our research uncovered a DNA demethylating activity of arsenic-containing Chinese herbal formula in the treatment of MDS.

## 1. Introduction

 Myelodysplastic syndromes (MDS) are characterized by functional abnormalities of hematopoietic lineages. Due to ineffective hematopoiesis, patients with MDS present with cytopenia(s) associated with morphological dysplasia and/or increase in number of blasts, and can progress to acute myeloid leukemia. The laboratory diagnostic strategy in MDS has evolved significantly over the years. Currently the integrated cytogenetic/genetic approach is critical for diagnosis and prognosis of MDS. DNA methylation is a biochemical process that is important for normal development in higher organisms. It involves the addition of a methyl group to the 5 position of the cytosine pyrimidine ring carried out by DNA methyltransferases. It has been shown that MDS patients, especially high-risk MDS, have abnormal hypermethylation of tumor suppressor genes [1]. 

 Our previous works indicated that Chinese herbal formula Qing-Huang powder (containing Realgar As_4_S_4_) is effective in the treatment of myelodysplastic syndrome (MDS) [2–4]. In the year 2011, we reported that of 124 cases of MDS patients treated with arsenic-containing herbal formula, complete remission and partial remission rate was achieved in 29 (23.4%) patients; hematologic improvement was achieved in 61 (49.2%) patients. The total efficacy rate was 80.6%. The clinical outcome after treatment was closely correlated with WHO Classification of myelodysplastic syndromes, MDS-risk scores, “International Prognostic Scoring System” (IPSS), and cyogenetic abnormality. However the underlined mechanisms remain unclear. To address the value of traditional medicine, efforts are required to delineate how the clinically effective formulae work at the molecular, cellular, and organism levels. 

 In this paper, using standard cytogenetic analysis (G-banded) and global DNA methylation method (ChIP-on-chip assays), we aimed to determine the effect of arsenic-containing Chinese herbal formula on karyotype status, the genomic methylation level as well as the correlation between aberrant DNA methylation and chromosome aberrations.

## 2. Materials and Methods

### 2.1. Patients and Treatment Regimen

Twenty-five patients enrolled in this study were from outpatient clinics between 2009 and 2011 after giving informed consent. All patients were diagnosed according to WHO classification system (2008) and international prognostic scoring system (IPSS) [5]. The inclusion criteria: measurements of clinical response were defined according to standardized response criteria for myelodysplastic syndromes established by the International Working Group [6, 7]; patient had to be 18 to 75 years old; patients must not have received another investigational or approved therapy for MDS within 4 weeks of study enrollment; patients had no history of severe dysfunction of heart, liver, or kidney; all patients provided informed consent before this study. The exclusion criteria: patients had severe adverse effects during treatment; patients were allergic to tested drugs; patients were pregnant, breast-feeding, or had a mental disorder during treatment.

 All patients orally took arsenic-containing Qing-Huang powder capsule combined with Bupi Yishen Decoction for 6 months. Qing-Huang powder capsule 0.4 g/day (containing Indigo 0.24 g, and Realgar 0.16 g); Bupi Yishen Decoction once a day, consisting of Sheng-shu-di, Shan-yao, Shan-yu-rou, Dan-pi, Fu-ling, Ze-xie, Bu-gu-zhi, Tu-si-zi, Zhi-shou-wu, Tai-zi-shen, Bai-zhu, Sheng-jiang, Da-zao, Sang-ren, Nu-zhen-zi, and Han-lian-cao were added for the patient with symptoms of Yin deficiency; Suo-yang, Ba-ji-tian, Gu-zhi, Zhi-fu-pian were added for the patient with symptoms of Yang deficiency. 

### 2.2. Detection of Blood Arsenic Concentration

After treatment with Qing-Huang powder capsule, arsenic level in peripheral blood was detected by the coupling of high performance liquid chromatography (HPLC) with inductively coupled plasma mass spectrometry (ICP-MS) with the lowest detection limitation of 0.03 ug/L. 

### 2.3. Cytogenetic Analysis

 Cytogenetic analysis was performed on marrow aspirates according to the standard methods. Chromosome preparations were G-banded using trypsin and Giemsa, and karyotypes were described according to International System for Human Cytogenetic Nomeclature (ICSN) [8]. Major aberrant clone was confirmed by fluorescence in situ hybridization (FISH), probes including CEP8, D5S23-5q31, D7S522-CEP7, and D20S108.

### 2.4. DNA Methylation and Data Analysis

 Methylation detection was performed using Affymetrix USA GeneChip Human Promoter 1.0 Array according to the manufacturer's protocol (detailed protocol can be found at http://www.affymetrix.com/). Briefly, bone marrow mononuclear cells from MDS patients were collected before and after treatment. Methylated fraction of DNA was obtained using EpiQuik Methylated DNA Immunoprecipitation Kit (Promega). DNA target was PCR-amplified, fragmented, and terminally labeled. After hybridization, washing, and staining, the chip was scanned following the instructions. Conditions of chips before and after standardization were shown in supplemental Figures  1 and  2 (see Supplementary Material available online at doi:10.1155/2012/969476). 

 DNA methylation detection was achieved in all 25 patients before treatment, in 14 patients after treatment. DNA methylation information from 5 normal cases traced from NCBI public data Bank served as control <http://www.ncbi.nlm.nih.gov/>. Patient characteristics are listed in Table 1.

### 2.5. Data Analysis and Statistics

 All data were analyzed using two tools—Gene Ontology (GO) analysis and Pathway analysis—from Gene Ontology database and the KEGG database, respectively. 

Gene Ontology online database was established by the Gene Ontology Consortium in 2000. The Gene Ontology project is a major bioinformatics initiative with the aim of standardizing the representation of gene and gene product attributes across species and databases. The database can be used to search or predict disease-related aberrantly expressed gene/function [9, 10]. The KEGG database has been in development by Kanehisa Laboratories since 1995. KEGG is a collection of online databases dealing with genomes, enzymatic pathways, and biological chemicals. KEGG connects known information on molecular interaction networks, such as pathways and complexes (this is the Pathway Database), information about genes and proteins generated by genome projects (including the gene database), and information about biochemical compounds and reactions (including compound and reaction databases). The higher order functional information is stored in the Pathway Database, which contains graphical representations of cellular processes, such as metabolism, membrane transport, signal transduction, and cell cycle [11–15].

 GO analysis and Pathway analysis are two major tools based on the above two bioinformatics databases and are frequently used to analyze gene and gene function via gene Chip assay. GO analysis is more specific about single gene function, while Pathway analysis is more about networks of gene interactions in the cells, and integrating the two tools will yield better results on gene function analysis. 

 In this study, we used both GO analysis (based on Gene Ontology database) and Pathway analysis (based on KEGG database) to map the corresponding genomic position and function. Statistical significance of the difference between values of methylation levels for different samples/groups were assessed using Fisher and multiple comparison test with *P* < 0.05, false discovery rate (FDR) < 0.05 as filtering cut-off point.

## 3. Results

### 3.1. DNA Methylation before Treatment

 According to conclusion drawn from GO analysis, 1063 hypermethylated genes were identified in 25 MDS patients compared with normal control, which consisted of 2.7% of the 4 million genes being tested. The hypermethylated genes involve 156 functional pathways, including response to stimulus, regulation of transcription DNA-dependent, multicellular organismal development, DNA-dependent positive regulation of transcription, signal transduction, apoptosis, cell cycle, and DNA repair, and so forth, as listed in Table 2 (parts of 156 functional pathways).

 Using Pathway analysis, we observed that 318 hypermethylated genes were identified in 25 MDS patients compared with control. The functions of these genes involve 46 different pathways, including pathways in cancer, Endocytosis, chemokine signaling pathway, ErbB signaling pathway, olfactory transduction, and so forth, as listed in Table 3 (parts of 46 functional pathways). 

 Consistent with multiple reports [16–23] and online database (http://www.ncbi.nlm.nih.gov/gene), our results indicate that MDS patients have aberrant DNA hypermethylation. Specifically, we confirmed that the following 27 genes, involving transcription control, signaling transduction, cell development, and adhesion, as well as cancer, are hypermethylated in MDS patients: ARNT2, E2F3, HDAC1, PIAS3, TCF7L1, TGFBR1, DCC, EPAS1, MET, WNT16, WNT6, WNT8B, FZD1, NCOA4, NFKB1, BCR, FASLG, FLT3LG, IGF1R, KIT, MAP2K1, PIK3R2, PLCG1, PLCG2, RALBP1, TRAF3, and SRC.

### 3.2. Karyotype and Gene Methylation

Among 25 MDS patients, normal karyotypes were observed in 12 cases, trisomy +8 was observed in 6 cases. The remaining 7 patients were cases of other type of aberrant karyotype, including 2 cases of −7, one case of 4p-, +9, dup(1q), −X/Xq-, and complex karyotype, respectively (Table 1). 

**Figure 1:**
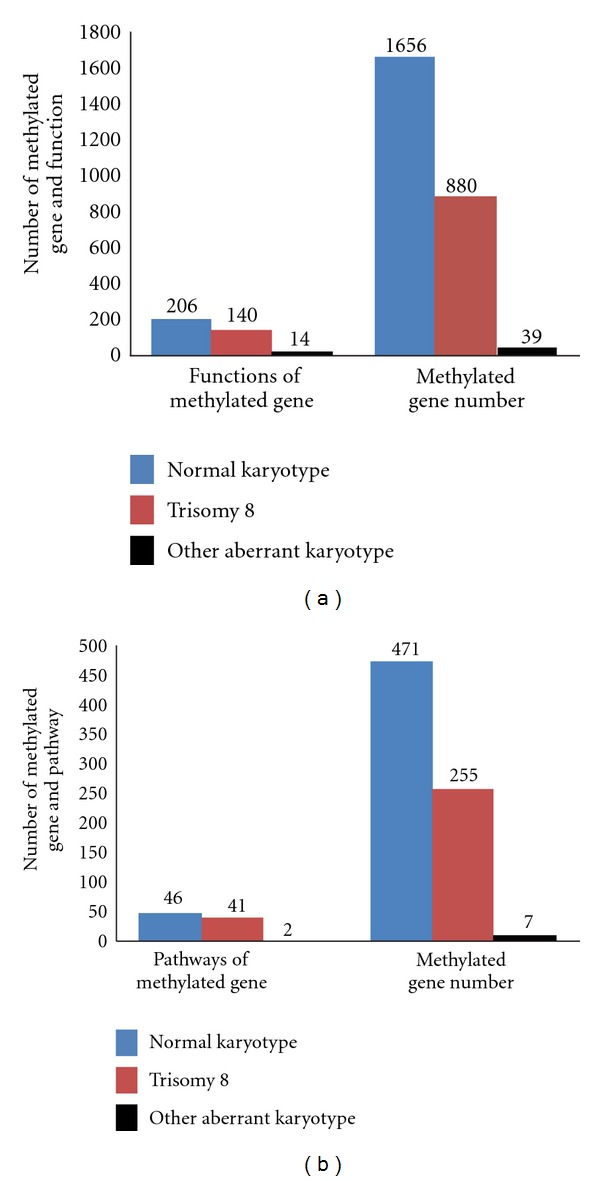


 Compared to healthy control, as analyzed by GO analysis, the number of aberrant DNA methylation is higher in patients with normal karyotype (1656 counts) and trisomy 8 karyotype (880 counts). DNA methylation is relative low in patients with cytogenetic abnormalities other than trisomy 8 (39 counts) (Figure 1(a)). The same DNA hypermethylation pattern is observed analyzed by Pathway analysis (Figure 1(b)).

### 3.3. Karyotype Status after Treatment

Certain amount of blood arsenic was observed in most patients (7.6 ug/L to 35.4 ug/L) after treatment. Hematologic responses were acquired after treatment with arsenic-containing Chinese herbal formula in 14 patients that have completed treatment for 6 months, including 2 CR and 12 hematologic improvements. Of these 14 patients, no karyotype change was observed after treatment in most cases, with the exception of one case, whose karyotype changed from 46, XX [20], trisomy +8 signal < 1.0% (by FISH) before treatment, to 47, XX, +8[6], -X[2]/46, XX[23], trisomy +8 signal > 40% (by FISH) after treatment. We concluded that arsenic-containing Chinese herbal formula had no obvious effects on karyotype status.

### 3.4. Demethylation Effects of Arsenic-Containing Chinese Herbal Formula

Six months of treatment with arsenic-containing Chinese herbal formula significantly decreased number of methylated genes and their functional signaling pathways in MDS patients.

 According to GO analysis tool, the number of hypermethylated gene decreased from 1063 to 75 after treatment. The functional pathways that involved gene hypermethylated decreased from 156 to 18 after treatment (Table 4). Further analysis indicated that the methylated genes involved in multicellular organismal development, signal transduction and apoptosis were demethylated after treatment.

 Analyzed by Pathway analysis tool, the number of hypermethylated gene decreased from 318 to 21 after treatment. The involved functional pathways of the hypermethylated genes decreased from 47 to 8 (Table 5). The methylated genes involved in cancer signals, chemokine signaling pathway, MAPK signaling pathway, calcium signaling, and so forth, were demethylated after treatment.

 Compared with multiple reports [16–23] and online database (http://www.ncbi.nlm.nih.gov/gene), our results indicate that 13 hypermethylated genes were demethylated after treatment (Table 6). Of these 13 demethylated genes, WNT8B, DCC, DACT1 involve multicellular organismal development; E2F3, NCOA4, HDAC1, PIAS3, ATF3, ZBTB33, LHX9 mainly involve transcription control; FASLG, TRAF3 involve cell apoptosis; PIK3R2 mainly involves signaling transduction. 

 Overall, our results indicated that after treatment with arsenic-containing Chinese herbal formula, the numbers of hypermethylated genes and the involved functional pathways were significantly decreased. The methylated genes involved in MDS pathogenesis were de-methylated after treatment with arsenic-containing herbal formula.

## 4. Discussion

 Aberrant DNA methylation is frequently observed in the myeloid malignancies, including myelodysplastic syndrome (MDS). With most researches focusing on single gene methylation [17–23], the limitations of these researches are obvious regarding global DNA methylation in MDS. In this study, using global DNA methylation method (ChIP-on-chip assays), we investigated genomic wide DNA methylation level in MDS thus reveal overall methylation profiling in MDS. We also determined the effect of arsenic-containing Chinese herbal formula on karyotype and the genomic methylation level in MDS patients.

 Our results indicated that among 4 million genes being tested 1063 genes were hypermethylated in MDS patients compared with control. The hypermethylated genes involve 156 functional pathways. Currently over 30 hypermethylated genes have been identified in MDS patients [16–23]. Confirmatively, we observed 17 of these hypermethylated genes in this study, namely FZD1, SOCS2, DKK1, DACT1, GADD45B, GADD45G, ARHGAP8, ARHGAP44, CDH7, ZBTB32, ZBTB33, ZBTB40, PAX2, ATF3, POU4F2, LHX2, and LHX9. The function of these genes is highly related to the pathogenesis of MDS. 

 After analyzing the correlation of DNA methylation status and karyotypes, we found that the number of genes with aberrant methylation is the highest in MDS patients with normal karyotype, higher in trisomy 8 karyotype, and relative low in patients with cytogenetic abnormalities other than trisomy 8 karyotype. Interestingly, we found that after treatment with arsenic-containing Chinese herbal formula, clinical outcome of MDS patients with normal karyotype or trisomy 8 is better than that of the patients with other cytogenetic abnormalities [4]. However arsenic-containing Chinese herbal formula had no effects on karyotype status of the patients.

 Furthermore, after treatment with arsenic-containing Chinese herbal formula, the number of hypermethylated genes decreased from 1063 to 75; the functional pathways affected by these hypermethylated genes decreased from 156 to 18. The methylated genes involved in multicellular organismal development, signal transduction, and apoptosis were demethylated after treatment. We identified 13 genes, whose function involving in multicellular organismal development, transcription control, signal transduction, apoptosis, cell development, and adhesion, as well as pathways in cancer, had been completely demethylated after treatment. The identified 13 demethylated genes including WNT8B, DCC, DACT1, E2F3, NCOA4, HDAC1, PIAS3, ATF3, ZBTB33, LHX9, FASLG, TRAF3, and PIK3R2), are critical for pathogenesis of MDS. Our research uncovered a novel DNA demethylating activity of Realgar-containing Chinese herbal formula in the treatment of MDS. 

 Currently DNA hypomethylating agents like the cytidine nucleoside analogs azacitidine and decitabine are widely used for the treatment of patients with myelodysplastic syndromes. Both drugs effected DNA methyltransferase-1 depletion and DNA hypomethylation [24–27]. However based on detoxification process of arsenic, we reason that arsenic hypomethylates DNA in a different pathway from azacitidine. Methylation of arsenic had been recognized as a detoxification process through methyltransferase enzymes using S-adenosylmethionine as methyl donor. Therefore the observed hypomethylating effects of arsenic in this study might be due to its competitive exhaustion of methyl during detoxification process of arsenic. Details regarding this mechanism are currently under investigation in the lab.

 In summary, in this study we found that aberrant DNA methylation correlates with MDS pathogenesis; the number of aberrant DNA methylation is highest in patients with normal karyotype, followed by trisomy 8 karyotype and relative low in patients with other cytogenetic abnormalities; treatment with arsenic-containing Chinese herbal formula resulted in a significant genome-wide demethylation, with no effects on karyotype status; the function of the demethylated genes is largely involved in transcription control, signal transduction, cell apoptosis, and multicellular organismal development. Our research uncovered a novel DNA demethylating activity of arsenic-containing Chinese herbal formula in the treatment of MDS. 

## Supplementary Material

Supplement Fig 1: Initial standardization of gene Chips: status before standardization (A) and after standardization (B).
Supplement Fig 2: Correlation of each pair of gene chips before standardization (A) and after standardization (B).

## Figures and Tables

**Table 1:** General information of patients.

Patient	Sex	Age	Diagnosis	Karyotype		Blood arsenic concentration (ug/L)	IPSS	Clinical evaluation
				Before	After			
1*	F	31	MDS-RCMD	47,XY,+8[10]	47,XY,+8[10]	7.60	Mid-risk I	Improve
2*	F	41	MDS-RCMD	46,XX,+8,−5[5]/47,XX,+8[15]	46,XX,+8,−5[15]/46,XX[1]	35.39	Mid-risk I	Improve
3*	M	31	MDS-RCMD	46,XY[20]	46,XY[20]	10.56	Mid-risk I	Improve
4*	M	23	MDS-RCMD	47,XY,+8[8]/46,XY[10]	47,XY,+8[11]/46,XY[9]	31.76	Mid-risk I	Improve
5*	M	41	MDS-RCMD	46,XY[20]	46,XY[20]	20.44	Mid-risk I	Improve
6*	F	25	MDS-RCMD	46,XX[20]	47,XX,+8[6],−X[2]/46,XX[23]	19.74	Mid-risk I	Improve
7*	M	26	MDS-RCMD	46,XY[20]	46,XY[20]		Mid-risk I	Improve
8*	M	34	MDS-RCMD	46,XY[20]	46,XY[20]		Mid-risk I	Improve
9*	M	48	MDS-RCMD	46,XY[20]	46,XY[20]		Mid-risk I	CR
10*	M	33	MDS-RCMD	46,XY[20]	46,XY[20]		Mid-risk I	Improve
11*	M	40	MDS-RCMD	46,XY[20]	46,XY[20]		Mid-risk I	Improve
12*	M	28	MDS-RCMD	46,XY[24]	46,XY[20]	11.42	Mid-risk I	Improve
13*	F	21	MDS-RCMD	46,XX[20]	46,XX[20]	18.34	Mid-risk I	CR
14*	F	57	MDS-RAEB I	45-46,−X/Xq-[10]	45-46-C/cq-(7?,9?,x?)[10]/46,xx[1]		Mid-risk II	Improve
15	F	31	MDS-RCMD	46,XX[16]			Mid-risk I	
16	F	57	MDS-RCMD	46,XX[20]			Mid-risk I	
17	F	25	MDS-RCMD	46,XX[8]			Mid-risk I	
18	M	40	MDS-RCMD-RS	47,XY,+8[6]/46,XY[1]			Mid-risk I	
19	M	43	MDS-RCMD	47,XY,+8[9]/46,XY[11]			Mid-risk I	
20	M	69	MDS-RCMD	45,XY, −7[16]/46,XY[3]			Mid-risk II	
21	F	36	MDS-RAEB I	46,XX,4p-[19]/46,XX[1]			Mid-risk II	
22	M	51	MDS-RCMD	48,XY,9q-,+10,+14[4]/9q-,+14[8]			Mid-risk II	
23	F	54	MDS-RCMD	47,XX,+9[16]			Mid-risk II	
24	M	34	MDS-RCMD	46,XY,dup(1q)[20]			Mid-risk II	
25	F	75	MDS-RCMD	45,XX, −7[13]			Mid-risk II	

*Gene methylation and karyotype were analyzed before and after treatment.

**Table 2:** Functions of genes with hypermethylation.

Go_id	Go_name	Diffgene _count	*P* value	FDR
GO:0050896	Response to stimulus	51	3.16*E* − 22	2.73*E* − 19
GO:0006355	Regulation of transcription, DNA-dependent	141	7.10*E* − 17	3.07*E* − 14
GO:0007275	Multicellular organismal development	105	2.50*E* − 16	7.23*E* − 14
GO:0045893	Positive regulation of transcription, DNA-dependent	59	7.24*E* − 11	1.57*E* − 08
GO:0007165	Signal transduction	115	1.58*E* − 10	2.73*E* − 08
GO:0000278	Mitotic cell cycle	37	5.86*E* − 08	7.84*E* − 06
GO:0006811	Ion transport	60	7.95*E* − 08	9.83*E* − 06
GO:0006915	Apoptosis	61	2.62*E* − 07	2.54*E* − 05
GO:0007264	Small GTPase mediated signal transduction	38	2.69*E* − 07	2.59*E* − 05
GO:0006810	Transport	59	4.52*E* − 07	3.58*E* − 05
GO:0006357	Regulation of transcription from RNA polymerase II promoter	31	4.58*E* − 07	3.61*E* − 05
GO:0016568	Chromatin modification	26	5.63*E* − 07	4.06*E* − 05
GO:0007049	Cell cycle	42	6.84*E* − 07	4.55*E* − 05
GO:0000122	Negative regulation of transcription from RNA polymerase II promoter	44	1.50*E* − 06	9.12*E* − 05
GO:0006281	DNA repair	33	2.47*E* − 06	1.36*E* − 04

This table shows parts of 156 category functions of genes with hypermethylation in 25 MDS patients. go_id: gene ID number in Gene Ontology database; go_name: category of function of gene; diffgene_count: number of gene methylated; *P* value: *P* < 0.01 indicating methylation is significant; FDR: false discovery rate, *P* < 0.05 as filter cutoff rate.

**Table 3:** Pathways of hypermethylated genes.

Path_id	Path_name	Diffgene _count	*P* value	FDR
5200	Pathways in cancer	41	2.22*E* − 05	1.15*E* − 03
5120	Epithelial cell signaling in *Helicobacter pylori* infection	14	1.11*E* − 04	2.86*E* − 03
3050	Proteasome	10	5.40*E* − 04	7.31*E* − 03
4144	Endocytosis	26	5.83*E* − 04	7.53*E* − 03
4062	Chemokine signaling pathway	24	1.20*E* − 03	9.49*E* − 03
4666	Fc gamma R-mediated phagocytosis	15	1.48*E* − 03	9.98*E* − 03
4650	Natural killer cell mediated cytotoxicity	19	1.75*E* − 03	1.03*E* − 02
5211	Renal cell carcinoma	12	2.13*E* − 03	1.07*E* − 02
5210	Colorectal cancer	11	2.54*E* − 03	1.09*E* − 02
4670	Leukocyte transendothelial migration	16	3.92*E* − 03	1.40*E* − 02
4612	Antigen processing and presentation	12	4.43*E* − 03	1.49*E* − 02
4360	Axon guidance	17	4.74*E* − 03	1.54*E* − 02
4012	ErbB signaling pathway	13	4.91*E* − 03	1.57*E* − 02
310	Lysine degradation	9	5.37*E* − 03	1.63*E* − 02
4740	Olfactory transduction	38	6.37*E* − 03	1.80*E* − 02

This table shows parts of 46 pathways of genes with hypermethylation in 25 MDS patients. Path_id: gene ID number in KEGG database; Path_name: pathway of gene; diffgene_count: number of gene methylated in specific pathway; *P* value: *P* < 0.01 indicating methylation is significant; FDR: false discovery rate, *P* < 0.05 as filter cutoff rate.

**Table 4:** Functions of genes with hypermethylation after treatment.

Go_id	Go_name	Diffgene _count	*P* value	FDR
GO:0007596	Blood coagulation	13	2.09*E* − 04	2.11*E* − 02
GO:0007088	regulation of mitosis	3	2.36*E* − 04	2.11*E* − 02
GO:0016337	Cell-cell adhesion	5	5.61*E* − 04	2.55*E* − 02
GO:0006357	Regulation of transcription from RNA polymerase II promoter	8	5.72*E* − 04	2.56*E* − 02
GO:0006325	Chromatin organization	3	1.11*E* − 03	3.05*E* − 02
GO:0006890	Retrograde vesicle-mediated transport, Golgi to ER	3	1.54*E* − 03	3.22*E* − 02
GO:0032312	Regulation of ARF GTPase activity	2	1.64*E* − 03	3.25*E* − 02
GO:0033690	Positive regulation of osteoblast proliferation	2	2.44*E* − 03	3.79*E* − 02
GO:0060710	Chorioallantoic fusion	2	2.44*E* − 03	3.79*E* − 02
GO:0006364	rRNA processing	4	3.02*E* − 03	4.05*E* − 02
GO:0043069	Negative regulation of programmed cell death	2	3.39*E* − 03	4.19*E* − 02
GO:0015031	Protein transport	9	3.50*E* − 03	4.22*E* − 02
GO:0005975	Carbohydrate metabolic process	8	4.92*E* − 03	4.56*E* − 02
GO:0006355	Regulation of transcription, DNA-dependent	20	5.43*E* − 03	4.65*E* − 02
GO:0007155	Cell adhesion	11	5.45*E* − 03	4.66*E* − 02
GO:0042953	Lipoprotein transport	2	5.75*E* − 03	4.70*E* − 02
GO:0048863	Stem cell differentiation	2	5.75*E* − 03	4.70*E* − 02
GO:0009395	Phospholipid catabolic process	2	7.14*E* − 03	4.87*E* − 02

This table shows functions of genes with hypermethylation after treatment. go_id: gene ID number in Gene Ontology database; go_name: category of function of gene; diffgene_count: number of gene methylated; *P* value: *P* < 0.01 indicating methylation is significant; FDR: false discovery rate, *P* < 0.05 as filter cutoff rate.

**Table 5:** Pathways of hypermethylated genes after treatment.

Path_id	Path_name	diffgene_count	*P* value	FDR
10	Glycolysis / Gluconeogenesis	4	5.97*E* − 03	3.82*E* − 02
4622	RIG-I-like receptor signaling pathway	4	8.21*E* − 03	3.82*E* − 02
5164	Influenza A	6	1.09*E* − 02	3.82*E* − 02
5215	Prostate cancer	4	1.82*E* − 02	4.34*E* − 02
4666	Fc gamma R-mediated phagocytosis	4	2.35*E* − 02	4.56*E* − 02
5131	Shigellosis	3	3.68*E* − 02	4.85*E* − 02
4964	Proximal tubule bicarbonate reclamation	2	3.71*E* − 02	4.85*E* − 02
5120	Epithelial cell signaling in *Helicobacter pylori* infection	3	4.90*E* − 02	4.99*E* − 02

This table shows pathways of hypermethylated genes after treatment. Path_id: gene ID number in KEGG database; Path_name: pathway of gene; diffgene_count: number of gene methylated in specific pathway; *P* value: *P* < 0.01 indicating methylation is significant; FDR: false discovery rate, *P* < 0.05 as filter cutoff rate.

**Table 6:** Demethylated genes profiles after treatment.

Go_id	Go_name	gene_symbol	*P* value	FDR
GO:0007275	Multicellular organismal development	WNT8B	1.05*E* − 09	2.69*E* − 07
		DCC	1.05*E* − 09	2.69*E* − 07
		DACT1	1.05*E* − 09	2.69*E* − 07
GO:0045893	Positive regulation of transcription, DNA-dependent	E2F3	1.12*E* − 07	1.15*E* − 05
		NCOA4	1.12*E* − 07	1.15*E* − 05
		HDAC1	1.12*E* − 07	1.15*E* − 05
GO:0006355	Regulation of transcription, DNA-dependent	PIAS3	3.24*E* − 06	2.08*E* − 04
		ATF3	3.24*E* − 06	2.08*E* − 04
		ZBTB33	3.24*E* − 06	2.08*E* − 04
GO:0006915	Apoptosis	FASLG	2.10*E* − 05	9.76*E* − 04
GO:0045892	Negative regulation of transcription, DNA-dependent	LHX9	2.32*E* − 05	1.04*E* − 03
GO:0006917	Induction of apoptosis	TRAF3	6.13*E* − 05	2.12*E* − 03
GO:0007165	Signal transduction	PIK3R2	2.58*E* − 04	6.45*E* − 03
