# Supplementary material for: Karyotype and DNA-Methylation Responses in Myelodysplastic Syndromes following Treatment with Traditional Chinese Formula Containing Arsenic
Source: Evid Based Complement Alternat Med. 2012 Oct 16;2012:969476. doi: 10.1155/2012/969476 (PMC3480675; doi:10.1155/2012/969476)
Supplement: Supplementary file 1 — Supplement Fig 1: Initial standardization of gene Chips: status before standardization (A) and after standardization (B). Supplement Fig 2: Correlation of each pair of gene chips before standardization (A) and after standardization (B). [file 969476.f1.pdf]

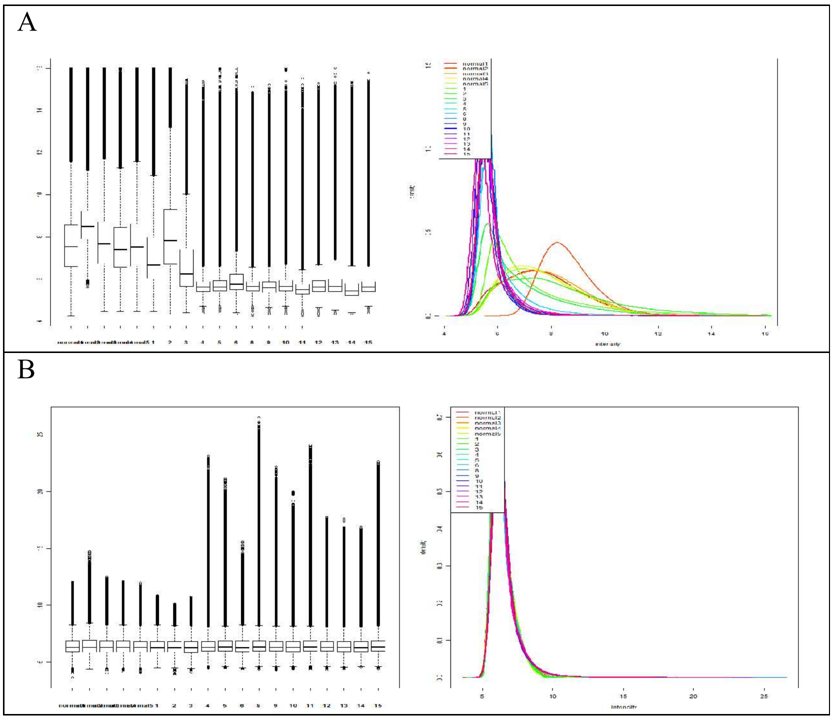


Supplement Fig 1 Initial standardization of gene Chips. A)Status before standardization; B) Status after standardization;

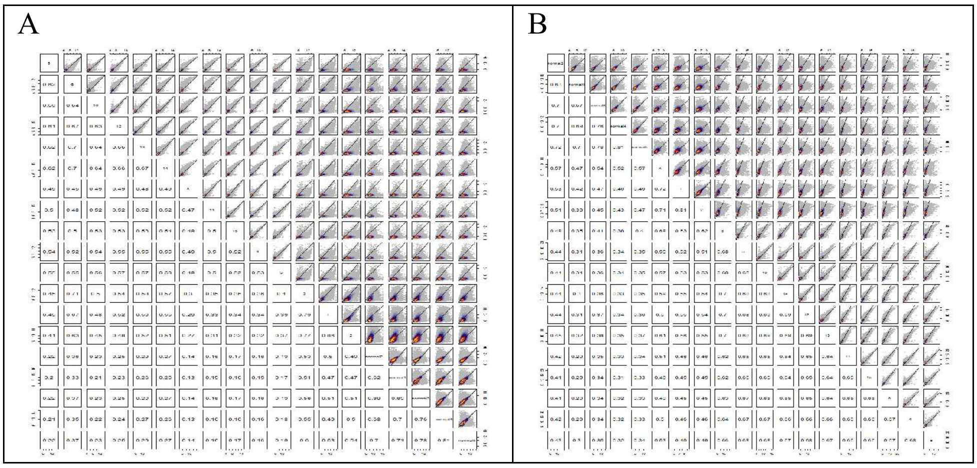


Supplement Fig 2. Correlation of each pair of gene chips before standardization (A) and after standardization (B). Scattered dots on top right corner represent signals from each pair of chips; Numbers in bottom left corner are correlation-ratio of each pair chips.
